# The key amino acid sites 199–205, 269, 319, 321 and 324 of ALV-K env contribute to the weaker replication capacity of ALV-K than ALV-A

**DOI:** 10.1186/s12977-022-00598-0

**Published:** 2022-08-24

**Authors:** Jian Chen, Jinqun Li, Xinyi Dong, Ming Liao, Weisheng Cao

**Affiliations:** 1 College of Veterinary Medicine, South China Agricultural University, 483 Wushan Road, Tianhe District, Guangzhou, 510642 China; 2 Key Laboratory of Zoonosis Prevention and Control of Guangdong Province, Guangzhou, 510642 China; 3 Key Laboratory of Zoonosis of the Ministry of Agriculture, Guangzhou, 510642 China; 4 Key Laboratory of Veterinary Vaccine Innovation of the Ministry of Agriculture, Guangzhou, 510642 China; 5 National and Regional Joint Engineering Laboratory for Medicament of Zoonosis Prevention and Control, Guangzhou, 510642 China

**Keywords:** Avian leukosis virus K subgroup, Env, Tva receptor, Recombinant chimaeras, Binding capacity

## Abstract

**Background:**

Avian leukosis virus (ALV) is an infectious retrovirus, that mainly causes various forms of tumours, immunosuppression, a decreased egg production rate and slow weight gain in poultry. ALV consists of 11 subgroups, A–K, among which ALV-K is an emerging subgroup that has become prevalent in the past 10 years. Most ALV-K isolates showed weak replication ability and pathogenicity. In this study, the weak replication ability of ALV-K was explored from the perspective of the interaction between ALV-K gp85 and the Tva receptor.

**Methods:**

Fourteen soluble recombinant ALV-A/K gp85 chimeric proteins were constructed by substituting the sequence difference regions (hr1, hr2 and vr3) of the ALV-A gp85 protein with the skeleton ALV-K gp85 protein for co-IP and competitive blocking tests.

**Results:**

The binding capacity of ALV-K gp85 to Tva was significantly weaker than that of ALV-A gp85 (P < 0.05) and the key amino acid sites 199–205, 269, 319, 321 and 324 of ALV-K env contributed to the weaker replication capacity of ALV-K than ALV-A.

**Conclusions:**

This is the first study to reveal the molecular factors of the weak replication ability of ALV-K from the perspective of the interaction of ALV-K gp85 to Tva, providing a basis for further elucidation of the infection mechanism of ALV-K.

**Supplementary Information:**

The online version contains supplementary material available at 10.1186/s12977-022-00598-0.

## Background

Avian leukosis viruses (ALVs), which represent a type of retrovirus responsible for various tumour diseases in chickens, are split into 11 subgroups: ALV-A to ALV-K [1–3]. ALV has caused great economic losses to the poultry industry, especially ALV-J, which has high tumorigenicity and affects the growth of broilers. As the purification process has progressed, a new subgroup of ALV, ALV-K, has been detected in China in recent years [4–7]. ALV-K isolates show weak replication ability and pathogenicity, which has helped ALV-K escape some company’s AL purification procedures and lurk in the chicken flocks for a long time. This has also led leading to a tendency of ALV-K to mutate or undergo recombination with other ALVs, resulting in the emergence of a virulent pathogenic strain of ALV-K and causing serious economic losses in the poultry industry [7–9].

ALV-K shares Tva receptors with ALV-A and invades the host [10, 11]; however, the replication ability of ALV-A is stronger than that of ALV-K [5, 6]. Our previous study showed that the long terminal repeat (LTR) promoter activity of ALV-K is weaker than that of other exogenous ALVs, which might be the reason for its low replication ability [12]. In addition, deletion of a few amino acids encoded by the *pol* gene leads to increased reverse transcriptase activity, which results in strengthened competitive replication advantages of ALV-K isolates [8]. When ALV-K isolates undergo recombination with other exogenous ALVs, their replication ability and pathogenicity are enhanced via the skeletons of other subgroups of ALVs [7, 9]. In this study, we focused on gp85 of ALV-A and ALV-K to explore whether the binding of these viruses to the Tva receptor also affects the replication ability of ALV-K.

The hr1 and hr2 regions of ALV-A–E gp85 are thought to be the key regions for receptor binding [3, 13–15]. In research on the functional domains of retrovirus envelope proteins, the viral envelope protein is usually replaced with an irrelevant sequence, and a recombinant chimeric protein is constructed for analysis of its binding capacity with the receptor protein. In addition, analysing the direction of virus mutation under the pressure of receptor immunoadhesins is also a common method to determine the functional domains of viral envelope proteins. Zhang et al. [16] determined the functional domain of the ALV-J gp85 protein by replacing the ALV-J gp85 protein segment with a segment including an HA-tagged protein with an unrelated sequence. Partial fragment deletion or mutation of the env of ALV-B and ALV-C occurred under the selective pressure of their immunoadhesins Tvb-mIgG and Tvc-mIgG. Thus, the mutated region was concluded to be the receptor binding region [14, 15]. In our previous study, we substituted the differential sequence of ALV-K and ALV-E (with Tvb as the host receptor[17]) and found that mutations occurring in the ALV-K envelope protein (env) at residues 194–198, 206–216 in hr1, 251–256 between hr1 and hr2, and 269–280 in hr2 affect its binding to the Tva receptor [18].

Under the action of soluble SUA-rIgG immunoadhesin blocking, ALV may alter the amino acids it binds to receptors on the cell surface, using cellular receptors from other subgroups of ALV to invade cells [19, 20], which provides a basis for the research idea of amino acid replacement between different subgroups of ALV membrane proteins. In our study, recombinant chimeric ALV gp85 proteins or recombinant chimeric ALVs were constructed by replacing the differential fragments in hr1, hr2 and vr3 regions of ALV-A and ALV-K, which barely affected the spatial structure of the ALV gp85 protein (Additional file 1: Fig. S1).

## Materials and methods

### Cell cultures and antibodies

293 T cells were cultured in Dulbecco’s modified Eagle’s medium (DMEM; Gibco, Life Technologies, Carlsbad, CA, USA) supplemented with 10% foetal bovine serum (BioInd, USA) in the presence of 5% CO_2_ at 37℃. DF-1 cells were cultured in DMEM with 10% FBS (Gibco, Life Technologies, Carlsbad, CA, USA) in the presence of 5% CO_2_ at 39℃. The anti-HA tag was purchased from Thermo Scientific (Rockford, IL, USA), whereas the anti-flag M2 tag antibodies and the anti-GAPDH antibodies were purchased from Sigma (Aldrich, St. Louis, MO).

### Comparative analysis of the replication ability of recombinant viruses

According to previous research methods [18], an RCASBP(A)-EGFP retrovirus was used as the vector to construct the point-mutated recombinant RCASBP(A)-EGFP retrovirus plasmid, and 1 µg of recombinant retrovirus were transfected into DF-1 cells in 6-well plates. After continuous passages, the cells were observed on the 3rd, 6th, and 9th days post transfection (DPT) by fluorescence microscopy. And the percentage of GFP positive cells was measured by flow cytometry after trypsin digestion. To analyze the replication competitiveness of ALV-A(RSA GenBank accession no. NC_001408.1) and ALV-K(GDFX0602 GenBank accession no. KP686143.1), recombinant viruses labelled with EGFP or mCherry were constructed, and 0.5 µg of each were cotransferred into DF-1 cells in 6-well plates. The cells were observed under a fluorescence microscope at 3, 6 and 9 DPT, and the percentage of GFP/mCherry positive cells were measured by flow cytometry after trypsin digestion.

### Expression of various chimeric soluble gp85 proteins

To identify the key amino acid residues of the ALV-A gp85 protein that interacts with Tva, gp85 of ALV-A RSA was cloned into the eukaryotic expression vector pCAGGS and fused with the 3 × flag tag sequence. To ensure that the gp85 protein was expressed in a soluble form, a fragment encoding a signal peptide (pCAGGS-s-gp85-flag) was fused with the N-terminus of the gp85 protein. A series of chimeric soluble gp85 proteins were constructed by replacing the corresponding sequence residues of RSA and GDFX0602 every five or six amino acids (Fig. 4 s1~vr3) by overlapping PCR, And the recombinant single-amino acid mutant gp85 proteins were constructed in the same way.

### Co-immunoprecipitation (co-IP) experiments and pull-down assay

Two hundred and ninety three T cells in 60-mm dishes were transfected with 5 µg of each respective chimeric gp85 plasmid using PolyJet (SignaGen Laboratories, Rockville, MD, USA) according to the manufacturer’s instructions. At 48 h after transfection, the supernatant of 293 T cells was collected and filtered through a 0.22-µM filter membrane, and then concentrated to 1/10 of the volume for co-IP experiments. For the binding assay in vitro, Tva was fused with the human IgG-Fc fragment, which specifically bound to the protein A/G of the plasmid pCAGGS-Tva-HA-Fc that was expressed in 293 T cells. The cell culture medium was collected, and the proteins were purified using protein A/G (Santa Cruz, Lexington, MA, USA) for 2 h at 4℃ with gentle agitation. After 5 washes with ice-cold phosphate-buffered saline (PBS), the agaroses were incubated with the cellular supernatant of 293 T cells transfected with the respective recombinant pCAGGS-s-gp85-flag and pCAGGS-Tva-HA-Fc for 6 h at 4 ℃ with gentle agitation. After 5 washes with ice-cold PBS, the bound proteins were separated by SDS-PAGE, and western blotting were performed using anti-HA, anti-flag and anti-GAPDH mAbs.

### Western blotting

High-temperature-denatured proteins were separated in 12% SDS-PAGE gels and transferred onto the NC membrane. After blocking in 5% (W/V) skim milk at room temperature for 1 h, an NC membranes were incubated with anti-HA mAb, anti-flag mAb and anti-GAPDH mAb at 4 ℃ overnight. After being washed 3 times with PBS, the NC membranes were incubated with IRDye^®^ 680RD donkey anti-mouse IgG (H + L) antibodies (LI-COR Biosciences, Lincoln, NE) for 1 h at room temperature. Finally, the NC membrane were scanned using an Odyssey Infrared Imaging System (LI-COR Biosciences).

### Competitive blocking test

To further verify the binding capacity between a series of recombinant gp85 series proteins and the Tva receptor, 10 µg of a series of recombinant pCAGGS-s-gp85-flag plasmids were transfected into 293 T cells in 100 mm culture plates. 48 h later, the cell supernatant was harvested and concentrated to 1/10 of the volume with an ultrafiltration tube, After filtrated with a 0.22 µm filter membrane, DF-1 cells were incubated with the recombinant gp85 protein after concentration at 4 ℃ for 1 h, and then infected with RCASBP(A)-EGFP virus. After 72 h, the cells were digested for calculating the percentage of GFP-positive cells by flow cytometry.

### Construction of recombinant virus mutants

To further verify the influence of the gp85 mutant fragments or single amino acids on Tva binding, RCASBP(A)-EGFP was used as the skeleton, a series of recombinant RCASBP(A)- EGFP-related viruses were constructed by overlapping PCR, and 1 µg of recombinant retrovirus were transfected into DF-1 cells in 6-well plates. After continuous passages, the cells were digested to calculate the percentage of GFP-positive cells by flow cytometry at 9 DPT.

### Construction of recovery mutants

At the protein levels, the amino acids identified by ALV-A gp85 directed mutation were replaced step by step with ALV-K gp85 as the skeleton to construct recovery mutants by overlapping PCR, and then co-IP assay was performed. At the viral levels, according to the idea of protein level restoration mutant construction, RCASBP(K)-EGFP was used as the skeleton to construct the recovery mutant recombinant virus and infected into DF-1 cells, After continuous passage, the percentages of GFP positive cells were counted by flow cytometry to evaluate the replication capacity of the recovery mutant recombinant virus.

### Analysis of ALV-A and ALV-K gp85 protein spatial structure

To evaluate the effect of segment by segment replacement of ALV-A and ALV-K gp85 sequences on the spatial structure of gp85 protein, I-Trasser (https://zhanggroup.org/I-TASSER/) was used to analyze the spatial structure of ALV-A gp85, and then ALV-A gp85 was used as a template to analyze the spatial structure of ALV-K gp85 by SWISS-MODEL (https://swissmodel.expasy.org/). And the spatial structure of ALV-A and ALV-K gp85 proteins were fitted by PyMOL software (Software version 2.2.0).

## Results

### Comparison of the replication ability of ALV-A and ALV-K

**Fig. 1:**
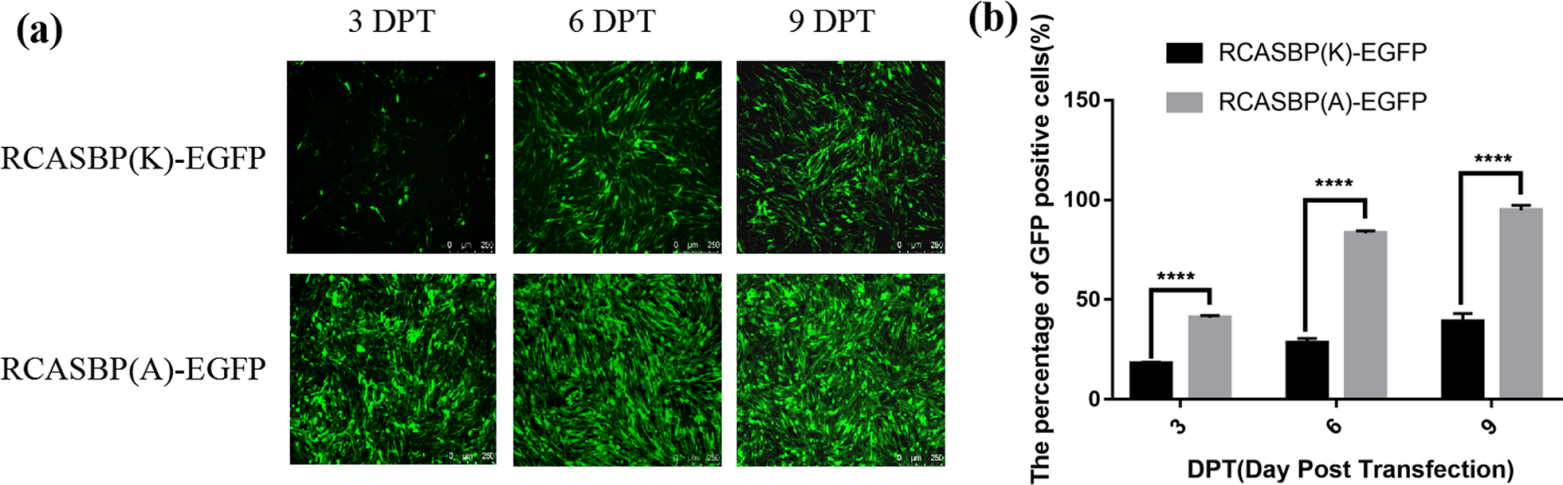
Comparison of replication capacity between RCASBP(A)-EGFP and RCASBP(K)-EGFP. **a** The replication capacity of RCASBP(A)-EGFP and RCASBP(K)-EGFP was observed by fluorescence microscopy. **b** The percentages of GFP-positive DF-1 cells transfected with RCASBP(A)-EGFP and RCASBP(K)-EGFP were determined by flow cytometry. Three independent experiments were performed, and the data are shown as the mean ± SD of triplicate samples from a representative experiment. Statistical analysis (two-way analysis of variance) was performed using GraphPad Prism 7; ****P < 0.0001

DF-1 cells transfected with RCASBP(A)-EGFP and RCASBP(K)-EGFP were continuously passaged, and fluorescence microscopy observation was performed at 3, 6 and 9 DPT. The results showed that DF-1 cells transfected with RCASBA-EGFP had high fluorescent signal intensity at 3, 6 and 9 DPT. The fluorescent signal intensity of RCASBP(K)-EGFP showed an obvious increasing trend after transfection, especially at 3 DPT, and the percentage of GFP positive cells transfected with RCASBP(A)-EGFP was significantly stronger than that of cells transfected with RCASBP(K)-EGFP (Fig. 1a, b, P < 0.001).

### Comparison of the competitive replication advantages of ALV-A and ALV-K

**Fig. 2:**
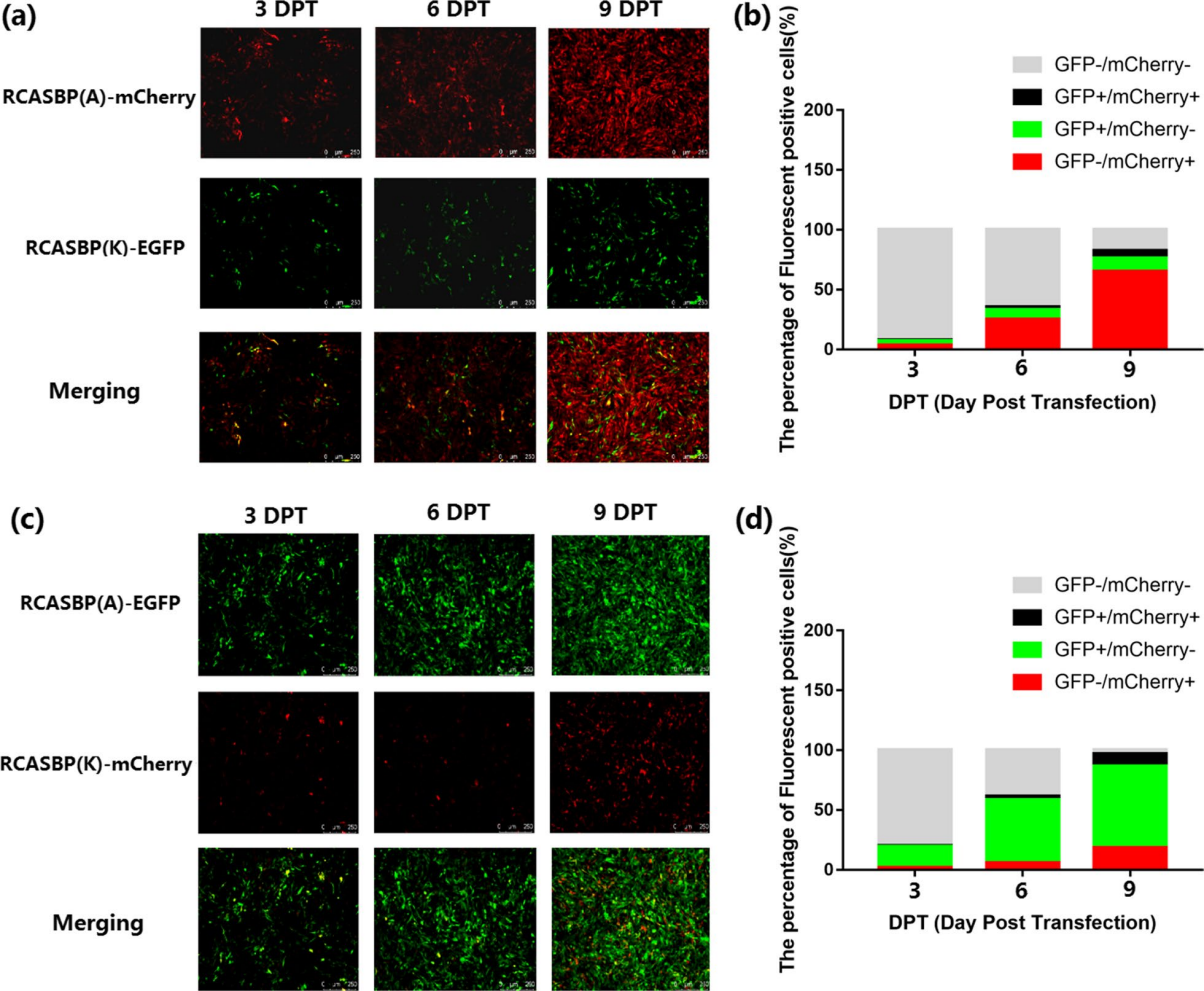
Comparison of replication competitive advantages between ALV-A and ALV-K based on RCASBP. **a**, **c** The replication capacities of RCASBP(A) and RCASBP(K) were observed by fluorescence microscopy. **b**, **d** The percentages of GFP/mCherry-positive DF-1 cells transfected with RCASBP(A)-EGFP and RCASBP(K)-EGFP were determined by flow cytometry. Three independent experiments were performed, and the data are shown as the means of triplicate samples

To evaluate the competitive replication advantages of ALV-A and ALV-K, which share Tva as the receptor, 0.5 µg of RCASBP(A)-EGFP/mCherry and 0.5 µg of RCASBP(K)-mCherry/EGFP were co-transfected into DF-1 cells in 6-well plates, respectively. After continuous passages, the fluorescence signal intensity was observed by fluorescence microscopy at 3, 6 and 9 DPT. The results showed that regardless of EGFP or mCherry labelling, the fluorescent signal of RCASBP(A) always had a dominant replication advantage (Fig. 2a–d).

### Comparison of the binding of ALV-A gp85 wt and ALV-K gp85 wt to the Tva receptor

**Fig. 3:**
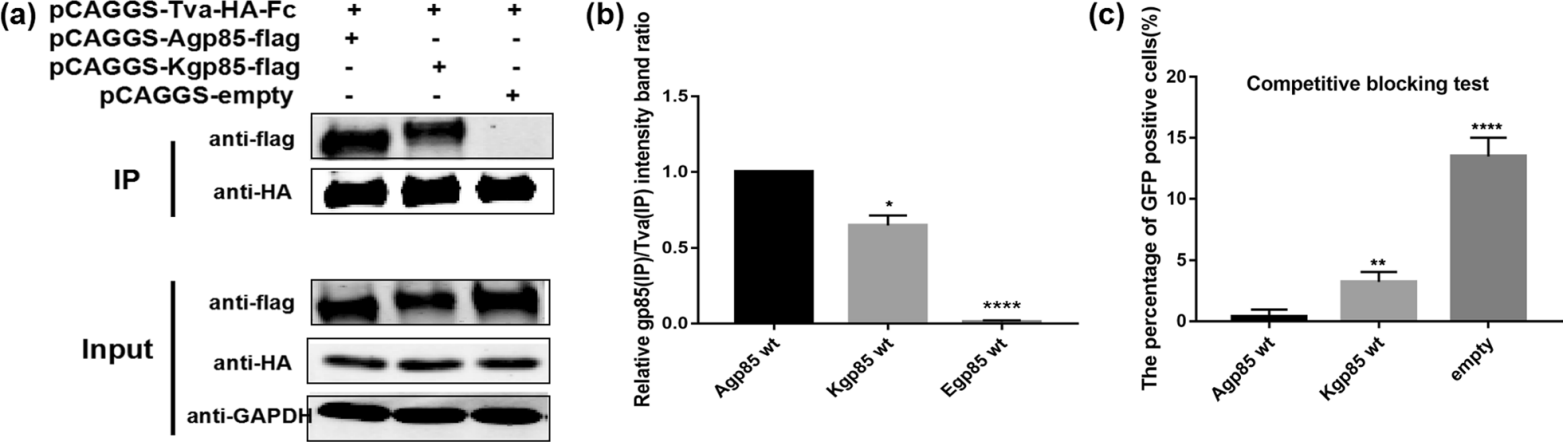
Analysis of the binding of the ALV-A and ALV-K gp85 proteins to the Tva receptor. **a**, **b** The co-IP test results and grey value analysis results for the ALV-A and ALV-K gp85 protein interactions with the Tva receptor. **c** Evaluation of the competitive blocking effect of the ALV-A and ALV-K gp85 proteins on RCASBP(A)-EGFP infection. Three independent experiments were performed, and the data are shown as the mean ± SD of triplicate samples from a representative experiment. Statistical analysis (two-way analysis of variance) was performed using GraphPad Prism 7; *P < 0.05, **P < 0.01, ***P < 0.001, ****P < 0.0001

To explore the effect of env on the difference in replication ability between ALV-A and ALV-K, the binding of ALV-A gp85 wt and ALV-K gp85wt to the Tva receptor were analyzed in this study. The co-IP results showed that the binding of ALV-K gp85 wt to Tva was significantly weaker than that of ALV-A gp85 wt (Fig. 3a, b, P < 0.05). Moreover, after transfection of 10 µg pCAGGS-s-A/K gp85 wt into 293 T cells in 100-mm plates and ten-fold concentration, ALV-A gp85 almost completely blocked the infection of DF-1 cells with RCASBP(A)-EGFP, but under the same conditions, about 3.0% of DF-1 cells were GFP-positive after blocking of ALV-K gp85 wt (Fig. 3c).

### Analysis of the binding of ALV-A/K gp85 with segmental replacement to Tva

**Fig. 4:**
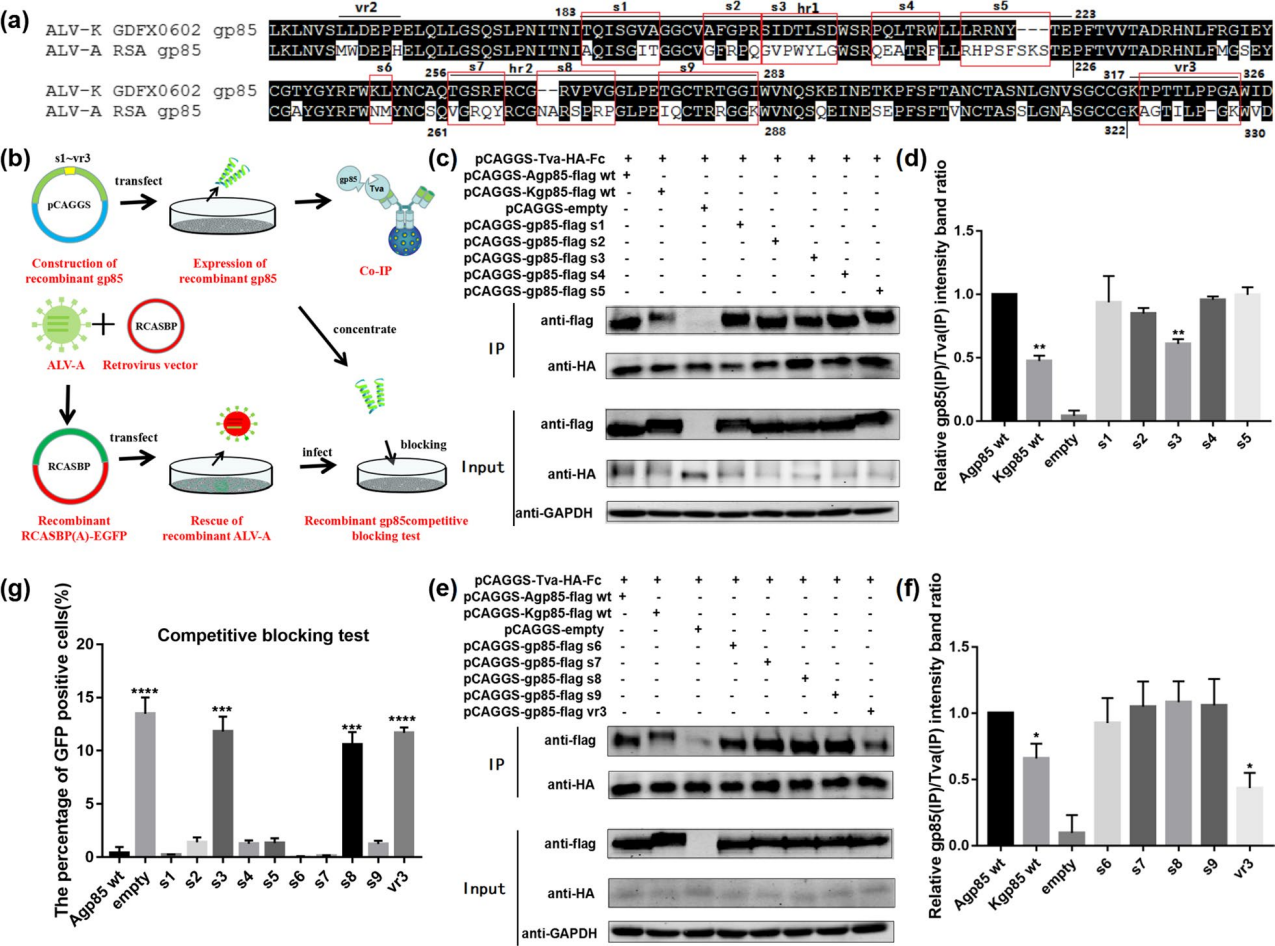
Analysis of the binding of a recombinant chimeric ALV-A/K gp85 protein for the Tva receptor. **a** Homology comparison between ALV-A RSA and ALV-K GDFX0602 gp85 protein sequences; **b** technology roadmap of co-IP and competitive blocking tests; **c**, **d** binding of recombinant chimeric fragments s1–s5 of the Agp85 protein to the Tva receptor and relative grey values; **e**, **f** binding of recombinant chimeric fragments s1–s5 of the Agp85 protein to the Tva receptor and relative grey values; **g** Competitive blocking of a recombinant chimeric ALV-A/K gp85 protein. Three independent experiments were performed, and the data are shown as the mean ± SD of triplicate samples from a representative experiment. Statistical analysis (two-way analysis of variance) was performed using GraphPad Prism 7; *P < 0.05, **P < 0.01, ***P < 0.001, ****P < 0.0001

To study the key amino acids in the differences in binding of ALV-A and ALV-K gp85 to the Tva receptor, the different parts of ALV-A and ALV-K gp85 hr1 and hr2 were divided into 10 segments (gp85 s1~vr3), and the corresponding fragments were replaced one by one (Fig. 4a). A series of soluble recombinant chimeric proteins were expressed for co-IP and competitive blocking experiments to analyse the binding of the soluble recombinant chimeric proteins to the Tva receptor (Fig. 4b). The co-IP results showed that the binding of the soluble recombinant chimeric proteins gp85 s3 and vr3 to the Tva receptor were significantly weaker than that of ALV-A gp85 wt (Fig. 4c–f, P < 0.01). In addition to the soluble recombinant chimeric protein gp85 s3 and vr3, DF-1 cells treated with the soluble recombinant chimeric protein gp85 s8 also showed about 11.0% GFP-positive, but the percentage of GFP-positive cells was slightly lower than that of DF-1 cells treated with the recombinant chimeric proteins gp85 s3 and vr3 (Fig. 4f). It’s worth noting that the soluble recombinant chimeric protein gp85 s8 was not identified in co-IP experiments; we speculated that the co-IP experiments were less sensitive than the competitive blocking experiments.

### Analysis of the binding of ALV-A/K gp85 s3 with single-amino acid replacement to Tva

**Fig. 5:**
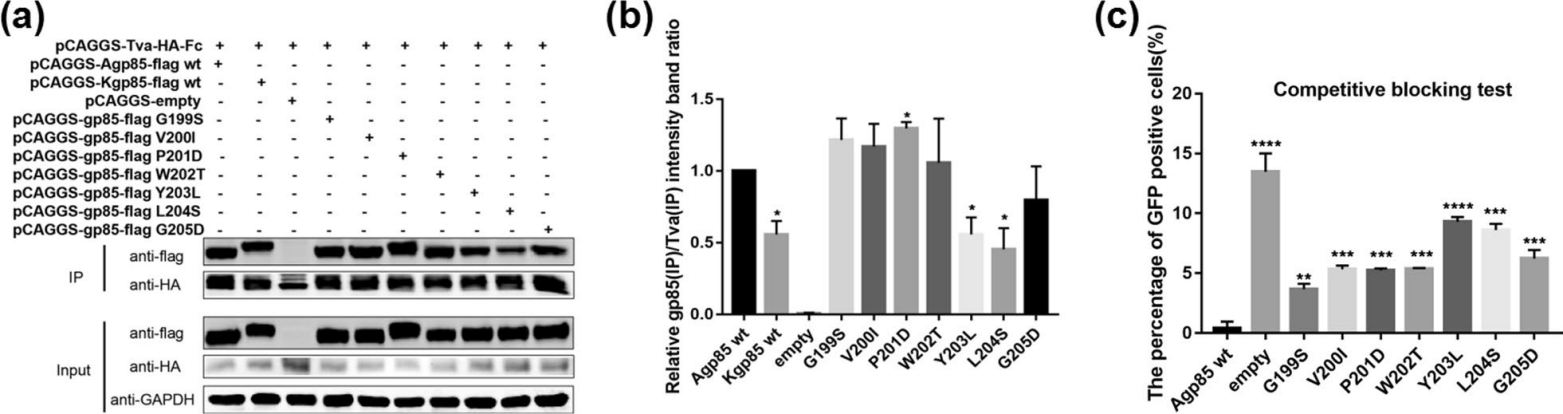
Analysis of the binding of recombinant gp85 s3 protein point mutants for the Tva receptor. **a**, **b** Co-IP test results and grey value analysis results for the interactions between soluble recombinant chimeric gp85 proteins and the Tva receptor; **c** evaluation of the efficiencies of soluble recombinant chimeric gp85 proteins in competitively blocking RCASBP(A)-EGFP infection. Three independent experiments were performed, and the data are shown as the mean ± SD of triplicate samples from a representative experiment. Statistical analysis (two-way analysis of variance) was performed using GraphPad Prism 7; *P < 0.05, **P < 0.01, ***P < 0.001, ****P < 0.0001

To further determine the key amino acids that cause the binding of ALV-A gp85 to Tva stronger than that of ALV-K gp85 to Tva, the amino acids of residue s3 were replaced one by one for the co-IP and competitive blocking experiments, the co-IP results showed that the binding of the single amino acid mutants Y203L and L204S to Tva was significantly weaker than that of ALV-A gp85 wt (Fig. 5a, b). When the DF-1 cell surface receptors were competitively blocked with soluble recombinant ALV-A gp85, the single-amino acid mutants Y203L and L204S showed low efficiency in blocking ALV-A infection of DF-1 cells, while the blocking efficiency of other single-amino acid mutants (G199S, V200I, P201D, W202T and G205D) were also lower than ALV-A gp85 wt (Fig. 5c), Therefore, all amino acids of s3 fragment affect the binding of ALV-A gp85 to the Tva receptor.

### Analysis of the binding of ALV-A/K gp85 s8 single-amino acid replacement mutants to Tva

**Fig. 6:**
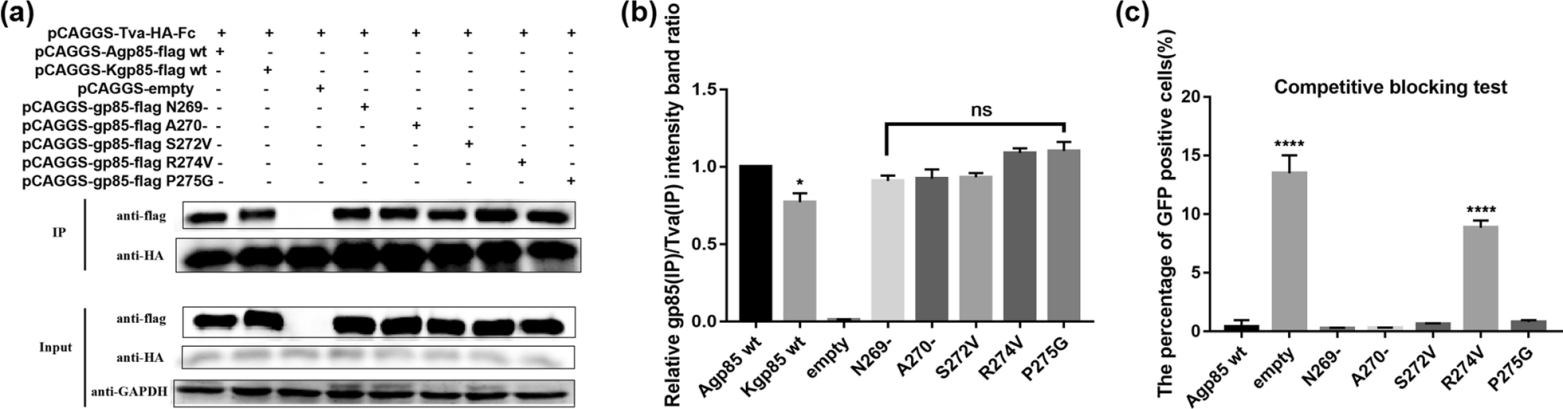
Analysis of the binding of recombinant gp85 s8 protein point mutants for the Tva receptor. **a**, **b** Co-IP test results and grey value analysis results for the interactions between soluble recombinant chimeric gp85 proteins and the Tva receptor; **c** evaluation of the efficiency of soluble recombinant chimeric gp85 proteins in competitively blocking RCASBP(A)-EGFP infection. Three independent experiments were performed, and the data are shown as the mean ± SD of triplicate samples from a representative experiment. Statistical analysis (two-way analysis of variance) was performed using GraphPad Prism 7; *P < 0.05, ****P < 0.0001, ns P > 0.05

To further determine the key amino acids that cause the binding of ALV-A gp85 to Tva stronger than the binding of ALV-K gp85 to Tva, the amino acids of residue s8 were replaced one by one. The co-IP results showed that there were no significant differences in binding capacity between recombinant the ALV-A gp85 s8 point mutants and Tva (Fig. 6a, b, P > 0.05). However, the results of the competitive blocking test showed that DF-1 cells corresponding to recombinant ALV-A gp85 R274V were about 9.5% GFP-positive (Fig. 6c), which was a higher percentage than that of GFP-positive DF-1 cells corresponding to recombinant ALV-A gp85 wt and other s8 point mutants. It is worth noting that ALV-A gp85 R274V was not identified in the co-IP test, which may have been because the sensitivity of the co-IP test was weaker than that of the competitive blocking test.

### Analysis of the binding of ALV-A/K gp85 vr3 single-amino acid replacement mutants to Tva

**Fig. 7:**
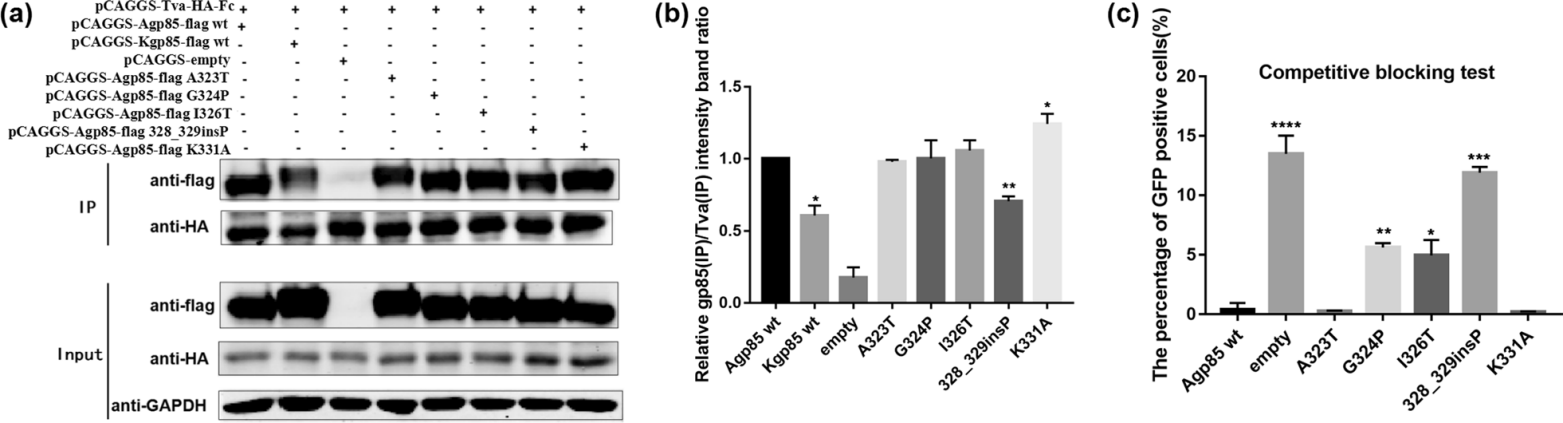
Analysis of the binding of recombinant gp85 vr3 protein point mutants for the Tva receptor. **a**, **b** Results of the co-IP test and grey value analysis for the interactions between soluble recombinant chimeric gp85 proteins and the Tva receptor; **c** evaluation of the efficiencies of soluble recombinant chimeric gp85 proteins in competitively blocking RCASBP(A)-EGFP infection. Three independent experiments were performed, and the data are shown as the mean ± SD of triplicate samples from a representative experiment. Statistical analysis (two-way analysis of variance) was performed using GraphPad Prism 7. Statistical analysis (two-way analysis of variance) was performed using GraphPad Prism 7; *P < 0.05, **P < 0.01, ***P < 0.001, ****P < 0.0001

To further explore the amino acids that affect the low binding capacity of recombinant ALV-A gp85 vr3 to Tva, the single amino acids of ALV-A and ALV-K env vr3 were substituted one by one to construct the vr3 point mutants. There was a single proline (Pro) insertion between sites 328 and 329 of ALV-A env, which was named mutant 328_329insP in this study. The results of the co-IP test showed that, the binding of the single-amino acid mutant 328_329insP to Tva was significantly weaker than that of ALV-A gp85 wt (Fig. 7, P < 0.01). The results of the competitive blocking test were essentially consistent with the results of co-IP, After DF-1 cells were blocked by the soluble recombinant ALV-A gp85 328_329insP and were infected with the recombinant virus RCASBP(A)-EGFP, about 12.5% GFP-positive DF-1 cells still existed. Compared with other single amino acid recombinant mutants, the soluble recombinant mutant ALV-A gp85 328_329insP showed the lowest blocking efficiency. In addition, there were still about 5.5% and 4.5% GFP-positive percentage in DF-1 cells corresponding to the single amino acid soluble recombinant mutants ALV-A gp85 G324P and I326T (Fig. 7c). The results showed that the ALV-A gp85 328_329insP mutant in vr3 greatly affected the binding of ALV-A gp85 to Tva. The point mutants G324P and I326T also affected the binding of ALV-A gp85 to Tva, but the effects were weaker than that of the point mutant 328_329insP.

### Viral reconstruction assay with point mutants

**Fig. 8:**
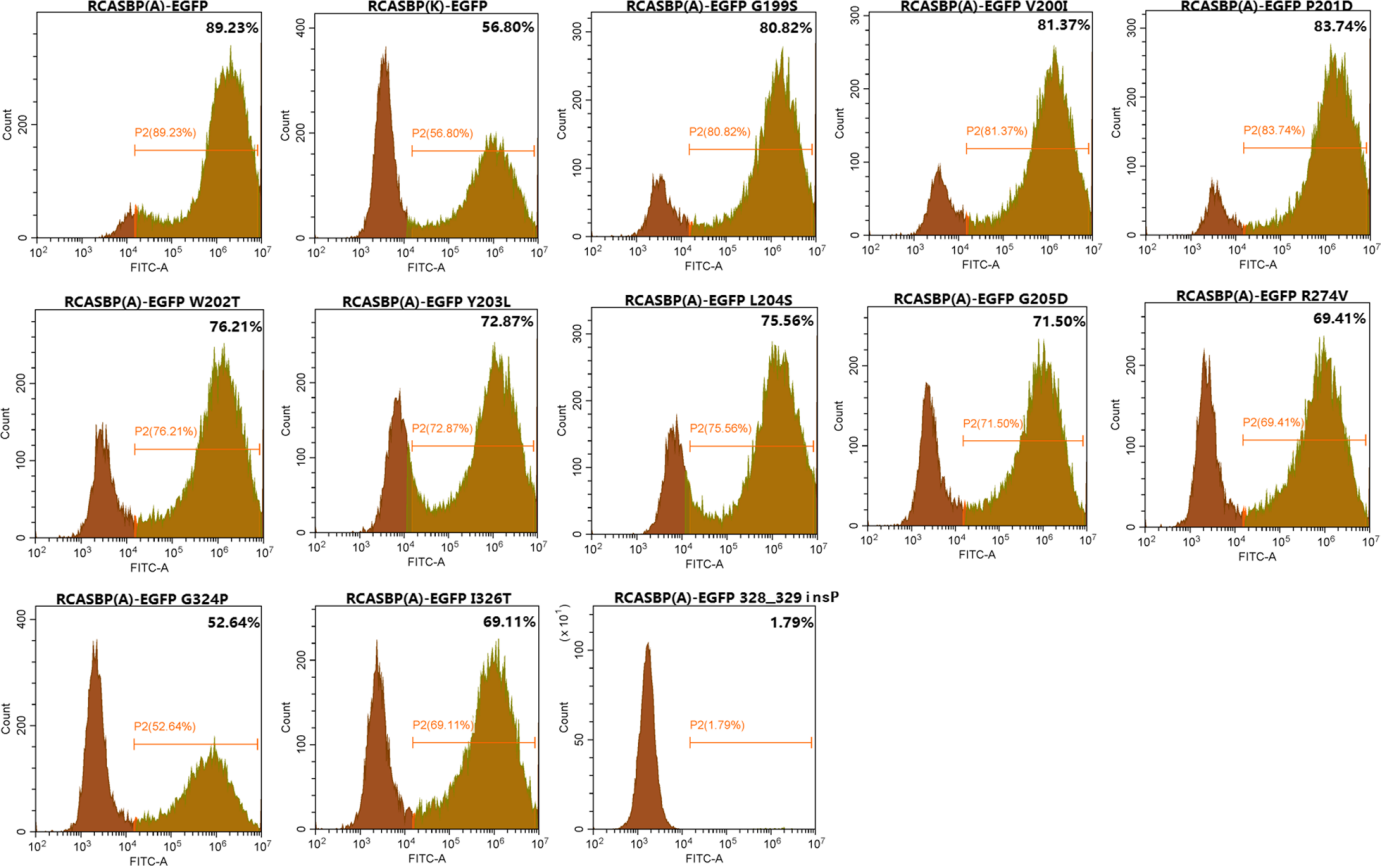
The percentages of GFP positive DF-1 cells fot the point mutants RCASBP(A)-EGFP G199S, V200I, P201D, W202T, Y203L, L204S, G205D, R274V, G324P, I326T and 328–329insP were analysed by flow cytometry

According to the previous construction methods, RCASBP(A)-EGFP was used as the skeleton to construct a series of point mutation recombinant viruses and transfected into DF-1 cells. After continuous passage, the percentage of GFP-positive cells was assessed by flow cytometry. The results showed that the percentages of GFP-positive DF-1 cells transfected with RCASBP(A)-EGFP G199S, V200I, P201D, W202T, Y203L, L204S, R274V, G324P, I326T and 328_329insP were lower than that of DF-1 cells transfected with RCASBP(A)-EGFP (Fig. 8). In particular, only 1.79% of DF-1 cells transfected with RCASBP(A)-EGFP 328_329insP were GFP-positive, which was a far lower than the percentage of GFP-positive cells transfected with RCASBP(K)-EGFP. However, aside from RCASBP(A)-EGFP G324P and 328_329insP, the percentages of GFP-positive cells among the cells transfected with other recombinant RCASBP(A)-EGFP viruses were higher than the percentage among the DF-1 cells transfected with RCASBP(K)-EGFP. This finding suggested that the replication ability of ALV-A was affected by ALV-A env G199S, V200I, P201D, W202T, Y203L, L204S, R274V, G324P, I326T and 328_329insP. In particular, the vr3 region point mutant 328_329insP greatly reduced the replication ability of ALV-A in vitro.

### Point mutation recovery experiment using ALV-K as a skeleton

**Fig. 9:**
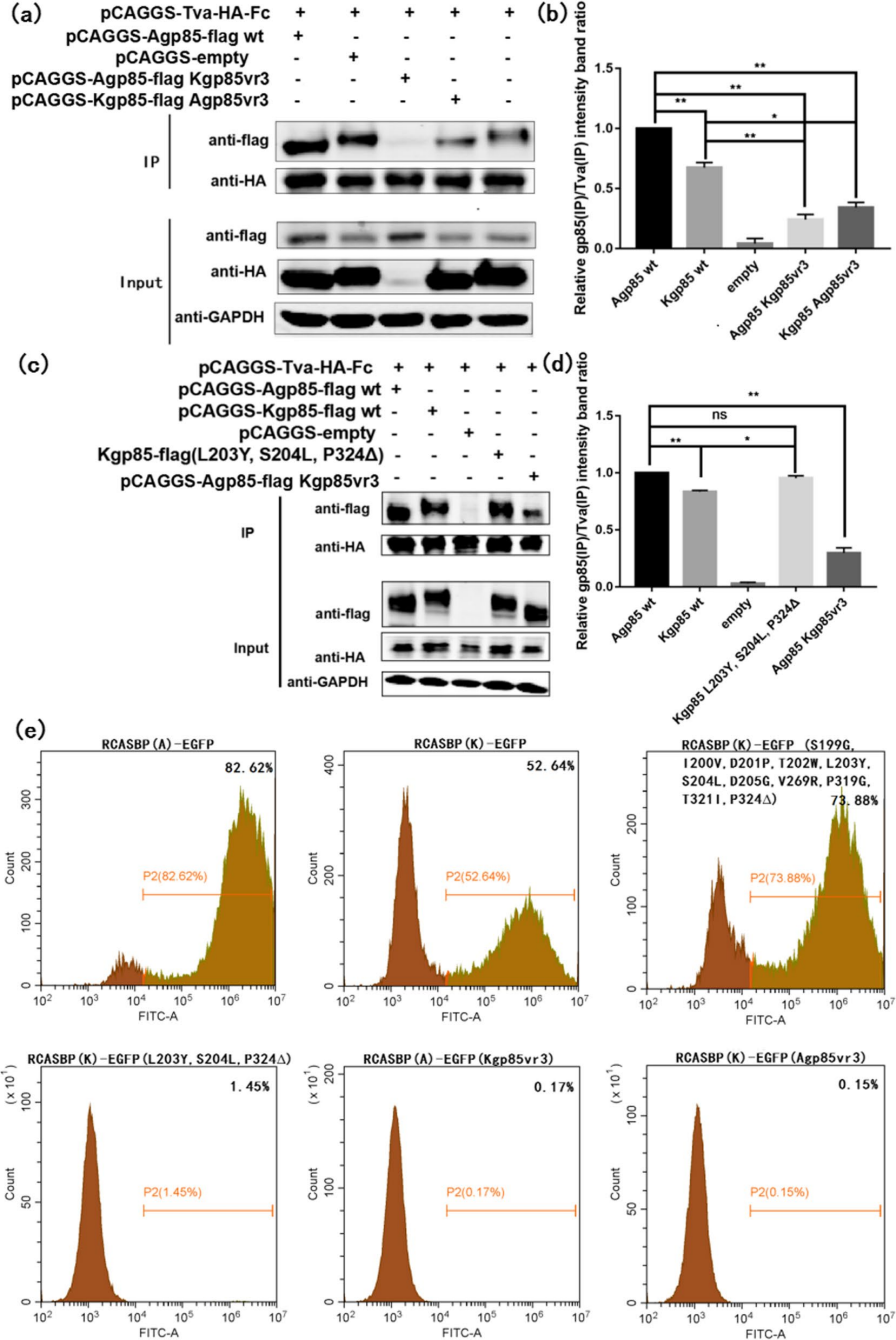
Recovery test of ALV-K gp85 protein point mutants. **a**, **b** Results of the co-IP test and grey value analysis of the interactions between soluble recombinant chimeric gp85 vr3 proteins and the Tva receptor. **c**, **d** Results of the co-IP test and grey value analysis of the interactions between restored soluble recombinant chimeric gp85 protein point mutants and the Tva receptor; **e** the percentages of GFP-positive cells for point mutation recovery mutants were analysed by flow cytometry. Three independent experiments were performed, and the data are shown as the mean ± SD of triplicate samples from a representative experiment. Statistical analysis (two-way analysis of variance) was performed using GraphPad Prism 7; *P < 0.05, **P < 0.01

Comparing to the amino acid sequences of ALV-A gp85 and ALV-K gp85, the identified key amino acid sites G199S, V200I, P201D, W202T, Y203L, L204S, G205D, R274V, G324P, I326T and 328_329insP of ALV-A gp85 corresponded to the amino acid sites S199G, I200V, D201P, T202W, L203Y, S204L, D205G, V269R, P319G, T321I and P324Δ of ALV-K gp85, respectively. To further verify the identified key amino acid sites causing the binding of ALV-A gp85 to Tva receptors, ALV-K gp85 protein sequences were used as the skeletons, and the co-IP tests were carried out. The results showed that the binding of the ALV-K mutants gp85 (L203Y, S204L and P324Δ) to the Tva receptor was similar to that of ALV-A gp85 wt to the Tva receptor (Fig. 9 c, d, P > 0.05), but significantly stronger than that of ALV-K gp85 wt to the Tva receptor (P < 0.05). This finding indicated that ALV env amino acid sites L203Y, S204L and 328_329insP were important factors affecting the stronger binding capacity of ALV-A gp85 than ALV-K gp85 for the Tva receptor. In addition, notably, the binding affinities of recombinant ALV-A gp85 (Kgp85 vr3) and ALV-K gp85 (Agp85 vr3) to the Tva receptor were weaker than that of ALV-K gp85 wt (Fig. 9a, b, P < 0.01). At the viral level, the percentage of GFP-positive cells of recombinant virus RCASBP(K)-EGFP (L203Y, S204L and P324Δ) was only 1.45%, however the percentage of GFP-positive DF-1 cells transfected with recombinant virus RCASBP(K)-EGFP (S199G, I200V, D201P, T202W, L203Y, S204L, D205G, V269R, P319G, T321I and P324Δ) was 73.88%, higher than 52.64% GFP-positive DF-1 cells transfected with RCASBP(K)-EGFP, but lower than 82.62% GFP-positive DF-1 cells transfected with RCASBP(A)-EGFP. The results showed that the sites 199–205, 269, 319, 321 and 324 of the ALV-K membrane protein were the key amino acid sites that caused the replication ability of ALV-K to be weaker than that of ALV-A.

### Discussion

The replication capacity and pathogenicity of ALV-K isolates were weaker than those of other exogenous ALVs. Previous studies have found that the promoter activity of ALV-K LTR is weak, which results in low replication titres of ALV-K [12]. Therefore, during the purification process of ALV, ALV-K is not easily detected, and can lurk in chickens for a long time, escaping the existing purification processes of some companies. In recent years, ALV-K isolates have been shown to undergo LTR recombination with other exogenous ALV LTR. Li et al. isolated a recombinant ALV-K strain, JS15SG01, with multiple ALV-K, ALV-E and ALV-J segments, containing the R3 and U5 regions of ALV-E LTR and the U3 region of ALV-J LTR; this strain caused increased levels of viremia and detoxification and caused brain tissue damage in chickens [9]. Lv et al. found that using ALV-J as skeleton and replacing the env with ALV-K env makes the replication capacity and pathogenicity of recombinant ALV-K were significantly stronger than those of the original ALV-K strain [7]. In addition to LTR, Su et al. [8] found that deletion of one amino acid at position 24 and eight amino acids at position 32–39 of ALV-K reverse transcriptase enhances viral replication in vitro and in vivo. Therefore, it is believed that the replication capacity of ALV-K is mainly affected by the ALV-K LTR promoter activity and reverse transcriptase activity. In this study, the percentage of GFP-positive DF-1 cells transfected with RCASBP(A)-EGFP was always higher than that of cells transfected with RCASBP(K)-EGFP. In addition, when DF-1 cells were further co-transfected with RCASBP(A) and RCASBP(K) carrying different labels in equal quantities, RCASBP(A) always showed stronger competitive advantages than RCASBP(K) (Fig. 2a–d). Subsequently, the binding affinities of ALV-A gp85 and ALV-K gp85 to the Tva receptor were compared in this study. The results showed that the binding capacity of ALV-A gp85 and the Tva receptor was significantly stronger than that of ALV-K gp85 (Fig. 3a, b), which was further supported by the competitive blocking of RCASBP(A)-EGFP infection by soluble ALV-A and ALV-K gp85 (Fig. 3c). Therefore, the binding of ALV-K env to the Tva receptor may also be a factor affecting ALV-K replication ability.

To further identify the key amino acids that affect the difference in binding affinities between ALV-A gp85 and ALV-K gp85 to the Tva receptor, a series of soluble recombinant chimeric ALV-A gp85 mutants were constructed with ALV-K gp85 as the control. In addition, the amino acid residues aa199–205 of hr1 of ALV-A env, aa269–275 of hr2 and aa323–331 of vr3 were determined to be crucial for the binding of ALV-A gp85 to Tva. Furthermore, a co-IP test and competitive blocking test were performed for the single amino acids sites 199–205, aa269–275 and aa323–331 of the ALV-A env. The results of the co-IP test showed that the amino acid sites 203 and 204 in aa199–205 in hr1 and 328–329 in aa323–331 of vr3 were critical to the interaction between the ALV-A gp85 and the Tva receptor. In addition, the results of the competitive blocking test with point mutants revealed that all sites (G199, V200, P201, W202, Y203, L204 and G205) in residues 199–205 of hr1; R274 in residues 269–275 of hr2; and G324, I326 and 328_329insP in residues 323–331 of vr3 played important roles in competitively blocking RCASBP(A) infection according to the binding of the soluble recombinant ALV-A gp85 point mutants to DF-1 cell surface receptors. It is worth noting that the key amino acid sites of ALV-A gp85 binding to Tva identified by the co-IP test and the competitive blocking test are not completely consistent. Among them, amino acid sites 203, 204 and 328–329 of ALV-A env were identified in both the co-IP and the competitive blocking tests. In conclusion, all the amino acid sites identified by the co-IP test were identified in the competitive blocking test, which indicated that the competitive blocking test had higher sensitivity than the co-IP and could identify differences in protein grey values that were difficult to distinguish with the co-IP test.

Previous research has suggested that vr3 does not directly participate in the binding of ALV env to the host receptor, but plays an important role in coordinating hr1 binding to the host receptors [19]. However, that study targeted only a single amino acid mutation, K261E, unlike our study involving replacement of the entire vr3 region. Our results showed that the vr3 region played an important role in the binding of ALV-A gp85 for the Tva receptor, and the key amino acid change was identified as the insertion of Pro into residues 328–329 of ALV-A gp85 (Fig. 7a–c). The replication titer of RCASBP(A)-EGFP 328_329insP was greatly reduced at the level of the recombinant virus (Fig. 8). The soluble recombinant ALV-K gp85 (L203Y, S204L, P324Δ) was expressed by revertant mutation of the vr3 and hr1 regions of ALV-A gp85 with the corresponding key single amino acids. The recombinant ALV-K gp85 (L203Y, S204L, P324Δ) had a significantly stronger binding capacity to the Tva receptor than ALV-K gp85 wt (Fig. 9c, d, P < 0.05), and the binding capacity was close to that of ALV-A gp85 wt (P > 0.05). This further proves that vr3 regions are involved in the interaction between ALV-A gp85 and the Tva receptor.

Most ALV-K isolates are weak in replication and pathogenicity in vivo and in vitro, therefore they may not be detected easily in chicken flocks for a long time, which makes it possible for the isolates to escape the AL purification procedures of some breeding companies, and ALV-K may be able to mutate easily or recombine with other endogenous or exogenous ALVs to generate pathogenic ALV strains, which would bring new challenges to the purification of AL in China. From the perspective of the interaction between ALV gp85 and the Tva receptor, this study elucidates, for the first time, the molecular mechanism by which the difference in receptor binding affects ALV-K replication, providing a basis for further elucidation of the infection mechanism of ALV-K and refinement of the purification program of ALV on poultry breeding farms.

## Conclusion

In conclusion, this study found that the binding of ALV-K gp85 to Tva was significantly weaker than that of ALV-A gp85 (P < 0.05) and that the key amino acid sites 199–205, 269, 319, 321 and 324 of ALV-K env contribute to the weaker replication capacity of ALV-K than ALV-A. This represents the first report of the molecular basis of the weak replication ability of ALV-K from the perspective of binding between the ALV-K gp85 protein and the Tva receptor, providing a basis for further elucidation of the infection mechanism of ALV-K.

## Supplementary Information

**Additional file 1: Figure S1.** The spatial structure of ALV-A RSA gp85 and ALV-K GDFX0602 gp85 were analyzed by PyMOL software. Red area: hr1 Yellow area: hr2 Purple area: vr3.

**Additional file 2: ** Biosafety statement.

## Data Availability

Not applicable.
