# Supplementary figures and images for: The key amino acid sites 199–205, 269, 319, 321 and 324 of ALV-K env contribute to the weaker replication capacity of ALV-K than ALV-A
Source: Retrovirology. 2022 Aug 24;19:19. doi: 10.1186/s12977-022-00598-0 (PMC9400301; doi:10.1186/s12977-022-00598-0)

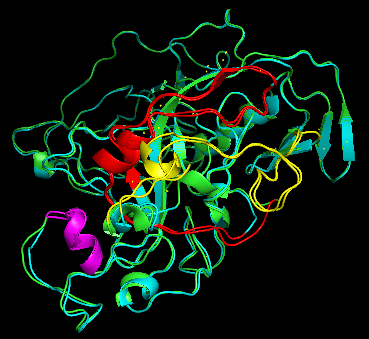

Supplement: Supplementary file 1 — Additional file 1: Figure S1. The spatial structure of ALV-A RSA gp85 and ALV-K GDFX0602 gp85 were analyzed by PyMOL software. Red area: hr1 Yellow area: hr2 Purple area: vr3. [file 12977_2022_598_MOESM1_ESM.png]
