# Supplementary material for: The key amino acid sites 199–205, 269, 319, 321 and 324 of ALV-K env contribute to the weaker replication capacity of ALV-K than ALV-A
Source: Retrovirology. 2022 Aug 24;19:19. doi: 10.1186/s12977-022-00598-0 (PMC9400301; doi:10.1186/s12977-022-00598-0)
Supplement: Supplementary file 2 — Additional file 2: Biosafety statement. [file 12977_2022_598_MOESM2_ESM.pdf]

Statement about the safety and the traceability of the series of recombinant viruses based on RCASBP vector

Retrovirology:

The manuscript "The key amino acid sites 199~205, 269, 319, 321 and 324 of ALV-K env contribute to the weaker replication capacity of ALV-K than ALV-A" submitted by Weisheng Cao et al. (Submission ID: d335525d-b3bc-4190-acb0-373bdd87b2f7), involved a series of recombinant viruses based on RCASBP vector. RCASBP vector is a kind of replication-competent avian retrovirus vectors containing the complete sequence of avian leukosis virus (ALV). According to the Chinese regulation on microorganism classification and laboratory safety, ALV is a kind of class III infectious microorganism in China with low risk of transmission and low pathogenicity, and has no known pathogenicity to all kinds of mammals and humans. All the authors of this study have solemnly made the following commitments:

1. All experiments in this study were approved by the biosafety committee of South China Agriculture University, and were conducted in accordance with relevant Chinese laws and regulations and under qualified laboratory conditions (such as ABSL-2, ABSL-3).

2. All the recombinant viruses are only for laboratory research and will not be released.

3. The sequences of all recombinant viruses are derived from the recombination of ALV-A RSA and ALV-K GDFX0602 and are clear (Fig.4a, in this manuscript). All the authors of this study have fully understood the relevant responsibilities and will earnestly fulfill the corresponding obligations and accept supervision and management.

陈建 李锦群 董欣怡 廖明

Authors: Jian Chen, Jinqun Li, Xinyi Dong, Ming Liao, Weisheng Cao

Jian Chen Jinqun Li Xinyi Dong Ming Liao Weisheng Cao

华南农业大学兽医学院

College of Veterinary Medicine, South China Agricultural University

Key Laboratory of Zoonosis Prevention and Control of Guangdong Province

May 6, 2022
